# Characterizing Sleep Phenotypes in Children With Newly Diagnosed Epilepsy

**DOI:** 10.1016/j.pediatrneurol.2022.07.016

**Published:** 2022-08-27

**Authors:** Temitayo Oyegbile-Chidi, Danielle Harvey, David Dunn, Jana Jones, Bruce Hermann, Anna Byars, Joan Austin

**Affiliations:** a Department of Neurology, University of California Davis, Sacramento, California; b Department of Public Health Sciences, University of California Davis, Sacramento, California; c Departments of Psychiatry and Neurology, Indiana University, Indianapolis, Indiana; d Department of Neurology, University of Wisconsin School of Medicine and Public Health, Madison, Wisconsin; e Department of Pediatrics, Cincinnati Children’s Hospital at the University of Cincinnati, Cincinnati, Ohio; f Distinguished Professor Emerita, School of Nursing, Indiana University, Indianapolis, Indiana

**Keywords:** Pediatric epilepsy, Behavior, Cognition, Sleep disturbance, Phenotype clusters

## Abstract

**Background:**

Children with epilepsy frequently have sleep, behavior, and cognitive problems at the time of or before the epilepsy diagnosis. The primary goal of this study was to determine if specific sleep disturbance phenotypes exist in a large cohort of children with new-onset epilepsy and if these phenotypes are associated with specific cognitive and behavioral signatures.

**Methods:**

A total of354 children with new-onset epilepsy, aged six to 16 years, were recruited within six weeks of initial seizure onset. Each child underwent evaluation of their sleep along with self, parent, and teacher ratings of emotional-behavioral status. Two-step clustering using sleep disturbance (Sleep Behavior Questionnaire), naps, and sleep latency was employed to determine phenotype clusters.

**Results:**

Analysis showed three distinct sleep disturbance phenotypes—minimal sleep disturbance, moderate sleep disturbance, and severe sleep disturbance phenotypes. Children who fell into the minimal sleep disturbance phenotype had an older age of onset with the best cognitive performance compared with the other phenotypes and the lowest levels of emotional-behavioral problems. In contrast, children who fell into the severe sleep disturbance phenotype had the youngest age of onset of epilepsy with poor cognitive performance and highest levels of emotional-behavioral problems.

**Conclusions:**

This study indicates that there are indeed specific sleep disturbance phenotypes that are apparent in children with newly diagnosed epilepsy and are associated with specific comorbidities. Future research should determine if these phenotypic groups persist over time and are predictive of long-term difficulties, as these subgroups may benefit from targeted therapy and intervention.

## Introduction

Sleep problems are commonly reported in children with epilepsy, regardless of epilepsy syndrome, and are often present at the time of diagnosis or even antecedent to the first recognized seizure.^1–3^ It is also well-documented that sleep and epilepsy are intricately interconnected. Several epilepsy syndromes (especially generalized genetic epilepsies) and neural sleep pathways share common thalamocortical networks, such that several neurotransmitters that regulate sleep also modulate seizures.^4,5^ As such, children with epilepsy often experience increased seizure frequency when sleep is not optimized. In addition, children with epilepsy are more likely to have seizures during sleep, especially during sleep-wake transitions, which can lead to significant sleep disruption.^1,6^ As a result, at least 45% of children with epilepsy have a sleep disorder.^2^

Children with epilepsy also frequently experience cognitive and behavioral problems, which are notable at the time of diagnosis.^3,7–9^ Few studies have directly investigated a relationship between poor sleep, cognitive problems, and behavioral/emotional problems in children with epilepsy; however, there is evidence that these comorbidities may be associated. Sleep plays a vital role in optimal cognitive performance, memory consolidation, and learning. As a consequence, disrupted sleep patterns in children with epilepsy may lead to poor memory, executive dysfunction, and other learning problems,^10–12^ as well as behavioral problems,manifesting as reduced attention span, impulsivity, and irritability/emotional lability,^13–17^ which present at higher rates than in children without epilepsy.^18^ All these findings suggest that the relationships among sleep, cognition, and behavior are interconnected, complex, and likely influence each other. Ultimately, these multiple comorbidities in children with epilepsy likely result in a lower quality of life.^17,19^

In the last several years, researchers have begun to appreciate the significant variability and heterogeneity in the risk of comorbidities among children and adults with epilepsy, and a move to identifying underlying latent groups of patients with varying comorbidity risk profiles (phenotyping) has proved to be helpful in this regard.^20^ Phenotyping focuses on identifying distinct groups of patients with epilepsy who cluster into particular risk categories based on specific profiles such as cognition and/or behavior. By clustering children with epilepsy into specific sleep disruption phenotypes, it may then become possible to identify more meaningful groups, their characteristics, and associated risks and comorbidities more accurately than examining the aggregate of patients as a whole.^21^ This approach may be more promising and lead to insights underlying the inconsistencies/variabilities reported in the relationships among sleep problems, cognition, behavior, and emotions in children with epilepsy.

The goal of this study was to characterize phenotypes of sleep problems in children with newly diagnosed epilepsy. In addition, we investigated the relationship of these phenotypes to baseline clinical epilepsy characteristics, cognition, and behavior. We hypothesize that there are discernible groups characterized by varying presence and severity of sleep problems and that the risk of cognitive and behavioral problems will covary with the sleep disturbance phenotypes. We further hypothesize that the sleep phenotypes will be characterized by differing baseline clinical epilepsy characteristics, which will vary across the groups and serve to tease out the inconsistencies that have been noted in the literature.

## Methods

### Participants

This study emanated from an investigation of children with new-onset seizures, their siblings as controls, and their primary caregivers.^22,23^ The core investigation was conducted at Indiana University and Cincinnati Children’s Hospital at the University of Cincinnati. A total of 354 children were recruited within six weeks of their first recognized seizure (mean = 35 days). Children were recruited through electroencephalogram (EEG) laboratories, emergency departments, and pediatric neurologists in two large children’s hospitals (Indianapolis and Cincinnati) and from practices of private pediatric neurologists in Indianapolis. All children in this sample had recurrent seizures during the duration of the study except for a total of 70 children. Of those 70 children who had only a single seizure during the duration of the study, 20 were started on medications soon after the commencement of the study, 18 had epileptiform activity recorded on EEG, and 13 had a history of multiple seizures before the beginning of the study. The sibling control sample was a comparison group of 266 healthy siblings of the children with epilepsy. Only one sibling was recruited per family.

Exclusion criteria for both children with epilepsy and siblings were a comorbid chronic physical disorder, intellectual disability (based on either clinic records or parent report), or seizures precipitated by an acute event (e.g., intracranial infection, metabolic derangement, and recent head injury). Children who had had two or more febrile seizures or who were placed on daily antiseizure medication after a febrile seizure were also excluded. The rationale for this latter exclusion was that the antiseizure medication might influence behavioral, emotional, or cognitive response to new-onset seizures. Parental informed consent and child assent were obtained before data collection. Siblings did not have epilepsy and were not on medication that could affect mental status. The study was approved by the institutional review boards at Indiana University and Cincinnati Children’s Hospital Medical Center.

Data were collected within six weeks of the first recognized seizure and focused on the time period six months before the seizure. Data were collected using computer-assisted, structured telephone interviews with the primary caregiver, who was the mother with very few exceptions.

### Instruments

#### Sleep evaluation

The Sleep Behavior Questionnaire (SBQ) was completed by the parent to characterize the child’s sleep problems during the prior six months. The SBQ has 35 items describing sleep habits and behaviors that are rated using five-point scales of 1 (*never*), 2 (*just a few times*), 3 (*sometimes*), 4 (*quite often*), and 5 (*always*). Parents were specifically instructed to exclude any behaviors that might have been actual seizure activity or any unusual sleep behaviors that occurred immediately before, during, or after a seizure episode. The reliability and validity of the SBQ as well as norms based on behavior and age have been established in the past.^2,14^ This study focused on the summary scores for bedtime difficulties, parent-child interactions, sleep fragmentation, parasomnia, and daytime drowsiness. The specific scales comprised in the summary scores are listed in Table 1. The final score varies between 26 and 130. The higher the score, the greater the number of sleep problems, which consequently indicates worse sleep disturbance overall. The SBQ was not obtained for sibling controls; however, published SBQ scores of healthy children around the same age and gender distribution were used for general comparison.

#### Cognitive evaluation

All children and sibling controls completed a comprehensive neuropsychologic test battery that included standardized clinical measures of intelligence, language, immediate and delayed verbal and visual memory, executive functions, speeded fine motor dexterity, and academic achievement. The specific tests administered included Clinical Evaluation of Language Fundamentals, 3^rd^ Edition^24,25^; Comprehensive Test of Phonological Processing^26,27^; Conners’ Continuous Performance Test, 2^nd^ Edition^28^; Kaufman Brief Intelligence Test^29,30^; Coding and Symbol Search Subtests of the Wechsler Intelligence Scale for Children, 3^rd^ Edition^31^; Wide Range Assessment of Memory and Learning (WRAML) Design Copy^32,33^; and the Wisconsin Card Sorting Test.^34–36^ Testing was administered by psychometrists who were trained, observed, and certified on the test battery and its scoring by a pediatric neuropsychologist.^36^

All instruments have high reliability and validity. Each test was administered according to the standardized procedures, and scores were converted to age-corrected standardized scores using the best available national norms for all tests except WRAML Design Copy, which was designed by this study’s research group and for which no norms are available. A prior factor analysis of this neuropsychologic test data^2,37^ revealed four underlying factors: (1) Language, (2) Processing Speed, (3) Executive Function/attention/construction (EF), and (4) Verbal Memory and Learning.^2^ The Language factor consisted of measures of verbal concept formation, phonological awareness, and phonological memory. The Processing Speed factor consisted of measures assessing psychomotor speed and rapid naming. The Executive Function (EF) factor consisted of measures assessing sustained attention, problem solving, and visual construction. The Verbal Memory and Learning factor consisted of measures of rote verbal learning and story recall.^2^ Higher factor scores indicate better neuropsychologic performance.

#### Behavioral evaluation

Three instruments were used to assess emotional and behavioral concerns—Child Behavior Checklist (CBCL), Children’s Depression Inventory (CDI), and Mean Affect Adjective Check List (MAACL).^38–40^ The CBCL was completed by a caregiver/parent to measure each child’s and sibling’s behavior problems during the past six months. Details of this instrument are provided elsewhere.^39^ Briefly, the CBCL has 118 items describing behaviors that are rated using 3-point scales of 0 (*not true*), 1 (*somewhat or sometimes true*), and 2 (*very true or often true*).^39^ For further information in regard to validity and reliability of the CBCL, see https://aseba.org/reliability-validity-information/. Three scores used in the study were the T scores for total behavior problems, internalizing problems, and externalizing problems, all of which are normed for age and gender. For the children with epilepsy, parents were specifically instructed to exclude any behaviors that might have been actual seizure activity or any behaviors that occurred immediately before, or after, a seizure episode. The CDI is a self-report questionnaire for children and adolescents designed to identify symptoms of depression in developmental age.^41^ The children with epilepsy completed this measure. The MAACL has been extensively used, well-validated, internally reliable, and shows good sensitivity to transient stressful conditions.^38,42^ We evaluated anxiety to assess overall psychological well-being. The children with epilepsy completed this measure.

### Statistical analysis

All data obtained were collated and analyzed using the Statistical Package for Social Sciences (SPSS) software (Version 27.0, IBM, Chicago, IL, USA). The SBQ variables included in the two-step clustering analysis were Bedtime Difficulties, Parent-Child Interactions, Sleep Fragmentation, Parasomnia, and Daytime Drowsiness, as well as Naps (yes/no) and Sleep Latency (in minutes). The two-step cluster analysis is a hybrid approach that initially capitalizes on a distance measure to separate groups and then a probabilistic approach (similar to latent class analysis) to select the optimal subgroup model.^43–45^ This technique offers several advantages when compared with more traditional clustering options, like determining the number of clusters based on a statistical measure of fit rather than on an arbitrary choice, using categorical and continuous variables simultaneously, analyzing atypical values (including outliers), and handling large datasets.^45^ Prior studies comparing clustering techniques regard two-step clustering as one of the most reliable in terms of the number of subgroups detected, classification probability of individuals to subgroups, and reproducibility of findings on clinical and other categories of data.^44,45^ The two-step cluster analysis was implemented in IBM SPSS Statistics (version 27.0). First, a sequential approach groups the cases based on the definition of dense regions in the analyzed attribute space. Second, a clustering approach statistically merges the grouped cases in a stepwise fashion until all groups of cases are sorted into specific clusters. Univariate analysis of variance was used to assess sleep phenotypes among children with epilepsy to evaluate differences in epilepsy characteristics, cognition, and behavior. Least significant difference was used for posthoc testing. Mixed effects analysis was employed to compare cognition and behavior scores in children with epilepsy and siblings. This statistical approach was used to address potential confounds of using sibling controls instead of typically developing healthy controls without an epilepsy sibling. All analyses controlled for age, gender, and number of medications.

## Results

### Demographics of the children with epilepsy (total epilepsy group)

Details of the demographics of this sample of children with epilepsy are listed in Table 2A. Briefly, the epilepsy sample consisted of 354 children with new-onset seizures aged six to 16 years. The majority of children were on medications, and the five most frequently prescribed medications were valproic acid, oxcarbazepine, carbamezapine, phenytoin, and lamotrigine. Other less commonly prescribed medications included felbamate, levetiracetam, phenobarbital, ethosuximide, topiramate, zonisamide and gabapentin.

Using mixed effects models to compare sibling controls with children with epilepsy, there were no significant differences in demographics; however, children with epilepsy had a lower intelligence quotient than siblings (98.16 vs 103.6) (*P* = 0.037). There were also significant differences in behavior and cognitive testing, indicating more behavior problems and poorer cognitive performance in children with epilepsy (Table 3B and C). In addition, overall, within the total epilepsy group (all children with epilepsy together), total sleep problems were remarkably higher among children with epilepsy compared with published controls (Table 3A).

### Characteristics of the sleep phenotypes

The two-step clustering analysis indicated that sleep phenotypes fell into a total of three clusters of *good clustering quality*. The three sleep phenotypes identified were Cluster 1 (minimal sleep disturbance, N = 185, 52.26%), Cluster 2 (moderate sleep disturbance, N = 60, 16.95%), and Cluster 3 (severe sleep disturbance, N = 109, 30.8%). Univariate analysis noted that each cluster group was significantly different from the others (Table 3A). In addition, the separate sleep problem categories also showed significant differences between all three clusters—bedtime difficulties (F(2,351) = 41.55, *P* < 0.001), parent-child interactions (F(2,351) = 72.75, *P* < 0.001), fragmented sleep (F(2,351) = 54.54, *P* < 0.001), parasomnias (F(2,351) = 36.64, *P* < 0.001), daytime drowsiness (F(2,351) = 17.32, *P* < 0.001), and sleep latency (F(2,351) = 29.85, *P* < 0.001) (Table 3A).

Notably, among the three sleep phenotypes, those with moderate and severe sleep disturbance showed twice as much sleep disturbance compared with published controls especially in para-somnias and daytime drowsiness. On the other hand, those with minimal sleep disturbance remarkably show a lower level of sleep problems in bedtime difficulties, parent-child interactions, and fragmented sleep compared with published healthy children (Table 3A).

### Behavioral problems in children with epilepsy by sleep phenotypes

Behavioral problems differed across sleep phenotypes of children with epilepsy such that children with minimal sleep disturbance exhibited behavior comparable to sibling controls and showed significantly less behavior problems compared with those in the moderate and severe sleep disturbance phenotypes (Fig B and C). Using the CBCL, internalizing problems (F(2,350) = 24.0, *P* < 0.001), externalizing problems (F(2,350) = 26.28, *P* < 0.001) and total behavioral problems (F(2,350) = 37.57, = *P* < 0.001) differed among the three sleep phenotypes (Table 3B). In addition, children with epilepsy with minimal sleep disturbance had significantly lower depression (CDI–F(2,243) = 4.35, *P* = 0.014) and anxiety (MAACL–F(2,351) = 17.61, *P* < 0.001) scores compared with those with moderate and severe sleep disturbance phenotypes (Table 3B).

### Cognition in children with epilepsy by sleep phenotypes

Cognition differed significantly across sleep phenotypes of children with epilepsy such that those with minimal sleep disturbance performed significantly better on all cognitive tests compared with those with moderate and severe sleep disturbance phenotypes (Table 3C). Of note, compared with sibling controls, cognitive scores were notably worse among all sleep phenotypes. Cognitive factor scores in Language (F(2,299) = 5.0, *P* = 0.004), Executive Function/attention/construction (F(2,299) = 5.92, *P* = 0.003), Verbal Memory/Learning (F(2,299) = 6.79, *P* = 0.001),and Processing Speed (F(2,299) = 6.24, *P* = 0.002) were significantly lower in moderate and severe sleep disturbance = phenotypes compared with the minimal sleep disturbance phenotype (Fig A).

### Clinical epilepsy characteristics by sleep phenotypes

Age, sex, race, handedness, and education showed no significant differences between clusters (Table 2A and B). There was, however, a significant difference in age of onset of seizures such that children who developed epilepsy at a younger age were more likely to experience severe sleep disturbance (F(2,351) = 3.64, *P* = 0.027). Furthermore, there were no significant differences among seizure burden (number of prior seizures), generalized versus focal seizure syndromes, seizure types, number of antiseizure medications, or use of attention medications. However, on general inspection, those with the severe sleep disturbance phenotype show the highest seizure burden and the highest percentage using attention medication (Table 2B). Other demographics (caregiver education and income) were also not different among phenotypes.

## Discussion

The main goal of our study was to determine if there are discernible phenotypes of sleep disturbance within a large cohort of children with epilepsy and to determine the relationship of identified sleep phenotypes and other epilepsy-related comorbidities. Indeed, our cohort fell into three distinct clusters based on the presence and pattern of sleep disturbance (minimal sleep disturbance, moderate sleep disturbance, and severe sleep disturbance) and co-occurred with the risk of multiple other comorbidities observed in children with epilepsy.

The findings of this study showed that children with a minimal sleep disturbance phenotype tended to have an older age of onset of epilepsy diagnosis and exhibited limited cognitive and behavioral concerns. Furthermore, children with this sleep phenotype had sleep disturbance levels similar to controls. Among all the children with epilepsy evaluated, the children within this subset had the best cognitive performance with the most intact scores in Language, Executive Function, Verbal Memory/Learning, and Processing Speed domains. This phenotype also showed behavioral rating scores (internalizing, externalizing, and total) similar to that of the control group, along with a low level of depression and anxiety; this suggests that the normal-appearing sleep phenotype may be more resilient to comorbidities in general, in spite of the epilepsy diagnosis. This finding of specific phenotypes in epilepsy being similar to control groups corroborates prior literature.^20^

On the other hand, children with a moderate or severe sleep disturbance phenotype had a younger age of onset of epilepsy diagnosis and exhibited the highest levels of cognitive and behavioral problems. By definition, children within these sleep phenotypes had the highest sleep disturbance levels. Among all the children with epilepsy evaluated, the children within this phenotype had the poorest cognitive performance with the lowest scores in Language, Executive Function, Verbal Memory/Learning, and Processing Speed cognitive domains. Furthermore, these phenotypes showed the highest levels of behavioral problems (internalizing, externalizing, and total) and emotional problems (depression and anxiety). Based on these findings, children with epilepsy who fall into these sleep phenotypes may be a subpopulation that should be targeted for potential therapy and therapeutics so as to improve quality of life.

This is the first study looking at sleep phenotypes in a large cohort of children with newly diagnosed epilepsy, demonstrating the presence of phenotypes of sleep disturbance with unique cognitive and behavioral signatures across each phenotype. Our study’s focus on a cohort with new-onset epilepsy is of specific importance as this shows that multiple comorbidities are already readily apparent right at the outset of the diagnosis of the disorder, which is counter to the classic view that comorbidities develop over a period of time. Our findings also suggest that a phenotype approach may be particularly illuminating as multiple comorbidities appear to co-occur together and also appear to pre-date the diagnosis of epilepsy. As a consequence, the adverse impact on quality of life may apparently become evident much earlier in the disease course than initially presumed. Therefore, our findings indicate the necessity for early identification of children at risk for comorbidities to improve quality of life sooner than later.

In the context of the available literature, one specific finding has remained consistent—i.e., only some children are affected by specific comorbidities, in terms of the presence and severity of these behavioral, cognitive, and sleep problems. For instance, depending on the specific study, 0% to 24% of children with epilepsy exhibit sleep problems,^46–49^ 25% to 47% of children with epilepsy exhibit cognitive problems requiring extra academic services at school,^10,16^ and 16% to 32% of children with epilepsy exhibit behavioral problems in the elevated range.^37,50,51^ These findings suggest that there is a group of children with epilepsy who are more resilient to these sleep, emotional-behavioral, and cognitive problems. In spite of these variabilities, there is no clear method at this time to predict who will and will not have sleep, cognitive, and behavioral problems. Capturing the nature and implications of this variable susceptibility to significant comorbidities is an important clinical and research topic.

The findings from this study especially indicate the interconnections among sleep, cognition, and behavior in children with epilepsy—the causal pathways across these comorbidities remaining to be determined. This study endeavored to begin to unravel these complexities in an attempt to further understand the origins of these problems and potential underlying modulators and moderators. Our findings reveal the considerable intertwined multimorbidity of pediatric epilepsy while using a phenotypic approach to distinguish those with high risk from others with low risk. Our findings also highlight the extensive challenge of determining a causal pathway among these multiple comorbidities as the individual sleep, cognitive, and behavioral problems primarily tend to aggregate together within specific subcohorts of children with epilepsy.

Conventionally, most studies in epilepsy tend to evaluate cognitive and behavioral comorbidities in isolation.^17–23,37,46–51^ However, the approach in our study uniquely exposed the considerable overlap among these individual epilepsy-related problems. This unveiled multicomorbidity factor needs to be more clearly defined with potential causal pathways identified and directly interrogated. Based on our findings, it is conceivable that abnormal sleep pathways may play a causative role and sleep problems might be involved in the development and persistence of the other comorbidities.^52^ However, our current study is primarily observational and as such has limited bearings on potential causal paths.

The inferences from our study are also limited as our controls were based on sibling data and published controls. Furthermore, some of the findings were based on subjective data (using well-validated surveys and self-evaluations). More advanced evaluations with polysomnogram and computational EEG analysis would be warranted in future studies to further understand the interrelationships of these multimorbidities using more objective measures. It is also important to note that antiseizure medications could have played a role in our findings as several antiseizure medications can affect sleep, behavior, and cognition adversely. However, the baseline findings are a reflection of sleep and behavior over the six months before the first seizure and cognition before or concomitant with starting antiseizure medications; this indicates that there is evidence of sleep, behavior and cognitive problems independent of antiseizure medications, which is consistent with prior literature that cognition and behavior may be abnormal before the diagnosis of epilepsy.^53,54^ In addition, a longitudinal assessment of sleep phenotyping in children with epilepsy and its associated comorbidities (cognition, behavior and emotional problems) along with causal modeling would be beneficial to further our understanding of the interconnections between these multiple comorbidities associated with pediatric epilepsy.

Overall, this study illuminates the role of sleep phenotyping in pediatric epilepsy to identify potential at-risk populations. Further research is necessary to determine the effect of treating these individual comorbidities on quality of life long-term in children with epilepsy.

## Figures and Tables

**FIGURE.**
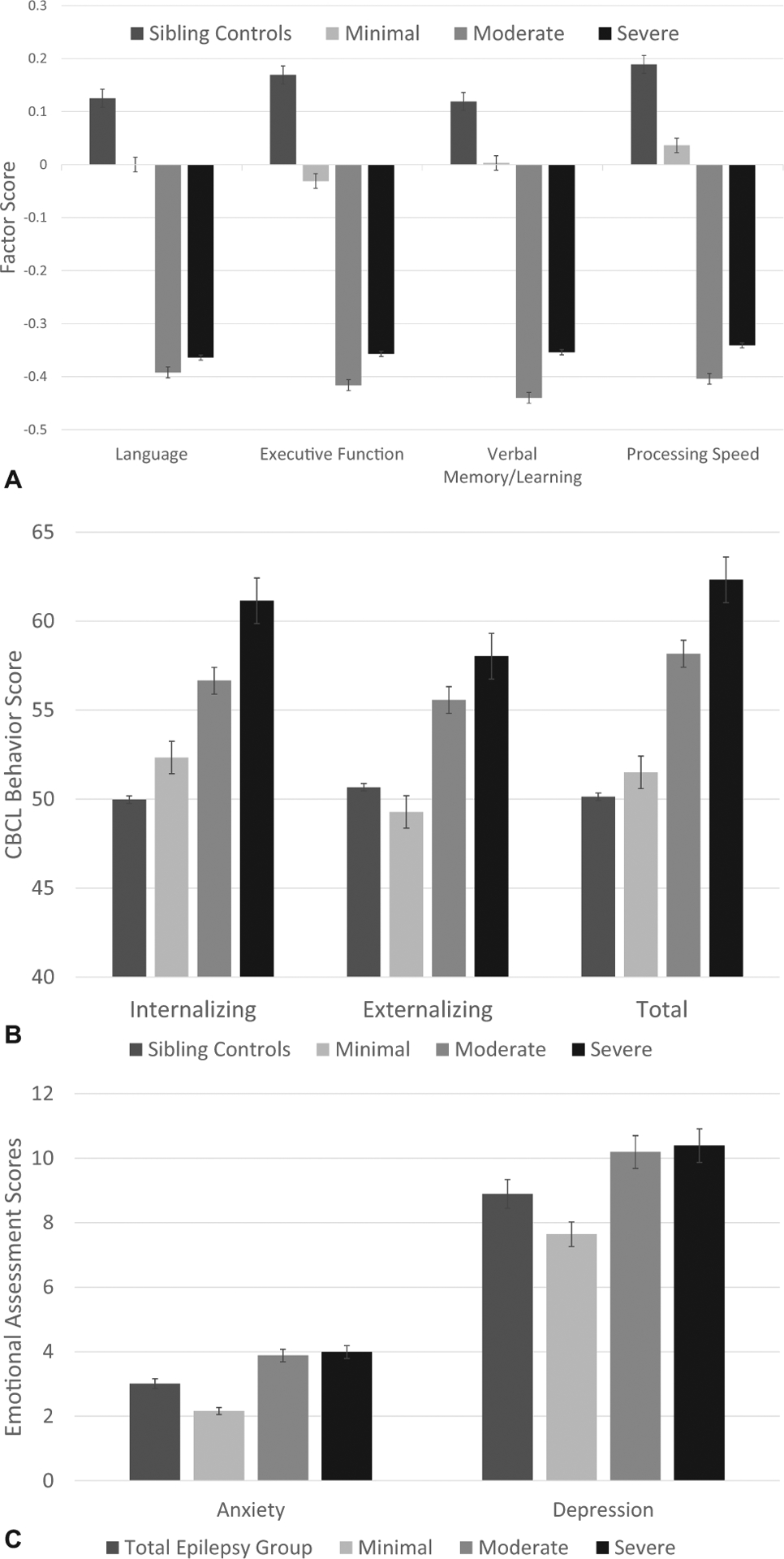
(A) Cognitive factor scores by sleep phenotypesd—minimal, moderate, and severe sleep disturbance phenotypes. Those who fall into the moderate and severe sleep disturbance phenotypes perform significantly worse on cognitive testing. (B) Behavior problems (CBCL) by sleep phenotypesd—minimal, moderate, and severe sleep disturbance phenotypes compared with sibling controls. Those who fall into the moderate and severe sleep disturbance phenotypes show significantly worse behavior.(C) Emotional-behavioral problems (CDI and MAACL) in children with epilepsy (total epilepsy group and among phenotypes). Children within the minimal sleep disturbance phenotype show significantly lower levels of anxiety and depression compared with moderate and severe sleep disturbance phenotypes. CBCL, Child Behavior Checklist; CDI, Children’s Depression Inventory; MAACL, Mean Affect Adjective Check List.

**TABLE 1.** Sleep Behavior Questionnaire Summary Score Categories

SBQ Summary Category	SBQ Specific Questions
Bedtime difficulties	Willingness to go to bed
	Sleeping alone
	Sleeping in his or her own bed
	Sleeping in parents’ bed.
Parent-child interactions	Night waking to go to parents’ bed
	Falls asleep again in parental presence
	Shares bedroom with parents
	Sleeps in parental bed
Sleep fragmentation	Wakes up 1 to 2 times per night
	Wakes up 3 to 4 times per night
	Wakes up for less than 30 minutes during the night
	Remains awake for more than 30 minutes during the night
	Wakes up to eat
Parasomnia	Sweats a lot during sleep
	Twitches while sleep
	Wakes up from sleep confused and disoriented
	Talks in sleep, walks in sleep
	Grinds teeth during sleep
	Wakes up from sleep screaming and terrified
Daytime drowsiness	Waking up refreshed and in a good mood
	Sleepiness while sitting or studying
	Sleepiness while watching TV
	Sleepiness while sitting and talking to someone
	Falling asleep at school

Abbreviations:

SBQ = Sleep Behavior Questionnaire

TV = Television

**TABLE 2.** Demographics of Sibling Controls and the Total Epilepsy Group as Well as Clinical Epilepsy Characteristics of the Three Sleep Phenotypes. (A) No Significant Differences in Demographics. (B) The Clinical Epilepsy Characteristics Indicate That Children With Younger Age of Onset of Epilepsy Are More Likely to Experience Severe Sleep Disturbance

	Sibling Controls	Total Epilepsy Group	Cluster 1: Minimal Sleep Disturbance	Cluster 2: Moderate Sleep Disturbance	Cluster 3: Severe Sleep Disturbance
A. Demographics					
Sample size	266	354	185	60	109
Age (y)	9.65	9.45	9.46	9.47	8.99
Sex M/F	128/138	166/188	85/100	26/34	55/54
B. Clinical epilepsy characteristics					
% R handed		80%	80%	82%	78%
Race (% white)		82.7%	84.2%	76.7%	86.2%
Age at onset		9.16	9.46	8.34	8.87*
Education		3.82	3.90	3.92	3.45
Seizure burden (S.E.)		76 (12)	50.9 (17.1)	82.2 (30.0)	105.2 (22.2)
% Focal seizures		56.3%	60.9%	56.1%	66.3%
Seizure types (%≤2 types)		13%	9.7%	20.7%	8.3%
% >1 Antiseizure medications		10.5%	18.4%	35.6%	6.6%
Attention medications (% on medication)		17.1%	12.9%	20%	24.1%
Caregiver education		13.81	13.98	13.41	13.42
Income ($)		60–70 k	60–70 k	60–70 k	60–70 k

Abbreviations:

F = Female

M = Male

R = Right

S.E. Standard error

* *P*< 0.05.

**TABLE 3.** (A) Breakdown of Sleep Phenotype Clusters (Published Controls From Cortesi et al., 1999^14^); Severe Sleep Disturbance (Cluster 3) Showed the Highest Level of Sleep Problems. (B) Behavior Problems in Sibling Controls and Children With Epilepsy (Total Epilepsy Group and Among Clusters). (C) Cognitive Testing in Sibling Controls and Children With Epilepsy (Total Epilepsy Group and Among Clusters)

	Controls Total Epilepsy Group		Cluster 1: Minimal Sleep Disturbance	Cluster 2: Moderate Sleep Disturbance	Cluster 3: Severe Sleep Disturbance
A. Sleep					
Total sleep problems	38.2	54.61	46.86	57.62*	66.22**
Bedtime difficulties	6.4	7.83	6.31	8.17*	10.14**
Parent-child interactions	8.4	7.45	5.44	7.57*	10.79**
Fragmented sleep	8.9	8.99	7.75	8.95*	11.15**
Parasomnias	8.9	12.31	10.61	12.96*	14.74**
Daytime drowsiness	5.5	9.77	9.16	11.42*	10.04**
Nap(%Y)			1%	99%	50%
Sleep latency (average mins)			16.37	19.47*	36.92**
B. Behavior					
CBCL internalizing	49.97	55.87	52.34	56.65*	61.14**
CBCL externalizing	50.67	53.15	49.28	55.57*	58.03*
CBCL total	50.13	56.09	51.50	58.17*	62.32**
CDI—depression		8.89	7.64	10.19*	10.39*
MAACL—anxiety		3.01	2.16	3.88*	3.99*
C. Cognition					
Language	0.125	−0.184	0.000*	−0.392	−0.364
Executive function	0.169	−0.185	−0.031*	−0.416	−0.357
Verbal memory/learning	0.119	−0.180	0.003*	−0.440	−0.354
Processing speed	0.189	−0.150	0.036*	−0.404	−0.341

Abbreviations:

CBCL = Child Behavior Checklist

CDI = Children’s Depression Inventory

MAACL = Mean Affect Adjective Check List

Y = Yes

For each row, Cluster 1 differs from Cluster 2 (*) and Cluster 3 (**). In addition, * significantly differs from **.

* *P* < *0.05*.
